# Are we closing the gender gap in academic oncology? An observational study of gender disparities in participant engagement at the ASCO 2024 annual meeting

**DOI:** 10.1136/bmjopen-2025-104821

**Published:** 2025-09-09

**Authors:** Hannah Christina Puhr, A Cammarota, M Ettaieb, Isabelle Flierman, T Gisinger, A Glas, Deniz C Guven, Alexander Siebenhüner, A Steindl, V Szydlik, S Valpione, M Yip, Hanneke W M van Laarhoven

**Affiliations:** 1 Department of Medicine I - Division of Oncology, Medical University of Vienna, Vienna, Austria; 2 Hepatobiliary Immunopathology Lab, IRCCS Humanitas Research Hospital, Rozzano (Milan), Italy; 3 Department of Biomedical Sciences, Humanitas University, Pieve Emanuele (Milan), Italy; 4 Amsterdam University Medical Centres, Amsterdam, Netherlands; 5 Department of Medicine III – Clinical Division of Endocrinology and Metabolism, Medical University of Vienna, Vienna, Austria; 6 Utrecht Medical Centre, Utrecht, Netherlands; 7 Department of Medical Oncology, Hacettepe University, Ankara, Turkey; 8 Klinik für Hämatologie und Onkologie, Hirslanden Zürich, Zürich, ZH, Switzerland; 9 Klinik für Gastroenterologie und Hepatologie, Universitätsspital Zürich und Universität Zürich, Zürich, Switzerland; 10 Faculty of Biology, Medicine and Health, The University of Manchester, Manchester, UK; 11 The Christie NHS Foundation Trust, Manchester, England, UK; 12 Cancer Research UK National Biomarker Centre, The University of Manchester, Manchester, UK; 13 Division of Cancer Sciences, The University of Manchester, Manchester, UK

**Keywords:** ONCOLOGY, Sexual and Gender Minorities, Health Equity

## Abstract

**Abstract:**

**Objective:**

Despite global efforts, gender disparities in oncology may persist. Understanding these disparities within the context of major conferences can inform strategies to promote gender inclusiveness in the field. This study evaluates the participation of women and men at the American Society of Clinical Oncology (ASCO) 2024 congress, focusing on chairs, speakers and audience questioners.

**Design:**

Observational study.

**Setting:**

152 recorded sessions of the ASCO 2024 annual meeting, one of the largest conferences in the field of oncology, available on the ASCO website.

**Participants:**

Individuals serving as chairs, speakers and audience members who asked questions.

**Primary and secondary outcome measures:**

In this observational study, gender for chairs, speakers and audience questioners across 152 sessions of the ASCO 2024 congress was assessed by two independent reviewers using audio and video recordings. Speaking times for questions and responses were also evaluated. Statistical analyses, including χ^2^ and unpaired t-tests, were conducted to analyse the data.

**Results:**

Women were well represented as chairs (n=124) and speakers (n=402) in 66% and 95% of sessions, respectively. However, only 21% of questions from the audience were posed by women, while 37% of questions were asked by men and 42% online or by chairs/speakers. Women were more likely to pose questions when the sessions were chaired by women (71% vs 53%; p=0.047). There were no statistically significant gender disparities concerning speaking time (questions: p=0.30; responses: 0.53). The response dynamics indicated a pattern of gender homogeneity, with individuals more frequently responding to questions from their own gender.

**Conclusions:**

While the balanced representation of women in leadership roles at the ASCO 2024 congress reflects positive development in gender equality, disparities in active participation persist. These findings underscore the need for strategies that not only promote women in visible roles but also foster an environment that supports their active engagement in scientific discussions.

**STRENGTHS AND LIMITATIONS OF THIS STUDY**

- Data were extracted from a large, internationally recognised oncology conference.
- Gender was assigned through systematic review of video recordings using consistent visual and auditory cues.
- Gender assignment was conducted by two independent reviewers and cross-checked by a third to ensure consistency and resolve discrepancies.
- The lack of self-reported demographic data limited the accuracy of gender and other participant characteristics.
- Analyses were restricted to a single congress, limiting generalisability across different settings or disciplines.

## Introduction

 In the USA, women constitute approximately one-third of all oncologists, highlighting a significant gender imbalance in the field.^1^ While the proportion in Europe appears more balanced, with over 50% of European Society for Medical Oncology (ESMO) members being women, persistent challenges to achieving true gender parity in the profession remain.^2^ The persistent pay gap, as well as limited opportunities for leadership roles and professional advancement for women compared with their male counterparts, exacerbates the existing disparities which, in fact, are not limited to the field of oncology.^3 4^ Women are less likely to receive recognition from professional societies as evidenced by their lower likelihood of receiving prestigious awards, with only 22% recipients between 1994 and 2019.^5^ Despite numerous initiatives (eg, Leadership Programme for Women in Oncology,^6^ Women Who Conquer Cancer,^7^ ESMO Women for Oncology (W4O)^8^ aiming to achieve gender parity, progress has been notably tardy as gender remains to act as a formidable barrier to career progression within oncology.^3 9 10^ In a survey conducted by the ESMO W4O Committee in 2021, one quarter of participants stated that gender had a significant or major impact on professional career opportunities, salary setting or related potential pay gap, while other factors such as ethnicity, sexual orientation and religion were considered to have little or no impact.^9^

Moreover, women’s participation in major oncology conferences as a presenter remains subdued. Between 2019 and 2023, only 23% of abstract presenters at ASCO and ASCO gastrointetsinal (ASCO GI) conferences were women regardless of presentation topic, region of presenter and meeting year.^11^ At the same time, women are less likely to actively engage as questioners in scientific discussions, and if they do, they talk for a shorter period of time than their male counterparts.^12 13^ Differential participation of women in significant scientific meetings might suggest that men continue to dominate the strategic directions of scientific discourse and decision-making within the field.

A study conducted at a large endocrinology conference suggests a direct link between the under-representation of women in leading positions and audience participation. It showed that increasing the number or visibility of women as chairs also increased the number of questions from women during scientific discussions.^14^ However, data from large oncology conferences are still lacking.

To further explore this dynamic, we analysed the roles of women in the American Society of Clinical Oncology (ASCO) 2024 congress, focusing on their participation as chairs, speakers and audience questioners. This analysis aims to shed light on current gender disparities at large academic congresses, thereby offering insights into the impact of existing barriers on the professional landscape of oncology.

## Materials and methods

### Data source

In this study, the gender disparities among chairs, speakers, questioners and respondents at the ASCO 2024 Congress were investigated. This conference was chosen for this analysis as it is a global platform in oncological scientific discourse and provides online access to recorded discussions. Therefore, recordings were sourced from ASCO’s official conference website^15^ (https://meetings.asco.org/meetings/2024-asco-annual-meeting), which were available to congress attendees.

As this analysis focused on sessions that had clear, recorded data on the questions posed during the sessions, we excluded sessions categorised as ‘Annual Business Meeting’, ‘Case-Based Panel’, ‘Highlights of the Day Session’ and ‘Poster Session’ from this analysis.

### Outcomes

Gender was defined as the categorisation (woman, man) perceived by the reviewers based on visual and auditory characteristics from the video recordings, such as names (ie, etymology and/or publicly available professional profiles), appearance (ie, attire, hairstyle, use of makeup) and voice characteristics (ie, pitch and tone). This method, though limited, is consistent with prior research.^12 13 16 17^ It has to be acknowledged that the assigned gender may not necessarily correspond to attendees’ self-identified gender.

Gender terminology in this study aligns with American Medical Association (AMA) guidelines.^18^

Speaking time was evaluated from the first sound until the last sound that was audible in the recording and could be distinctly attributed to a person and measured in seconds. Furthermore, in instances where question responses involved speakers of both genders, the response was classified as ‘answered by both genders’ and it was documented which gender responded first. The speaking duration was accordingly allocated to the respective genders. The average duration of a question and the response by each gender was calculated by dividing the total speaking time by the number of speakers or questioners of that gender.

### Data collection

Two independent reviewers assessed each session by evaluating the gender (women, men) of the chair/speaker/questioner based on given information (eg, name and institution), voice and video footage if available. Each occurrence of an individual serving as a participant was counted separately for each session, regardless of repeat appearances in multiple sessions. Discrepancies between the reviewers were resolved by a third reviewer who reassessed the contentious videos to determine the most plausible data set.

### Statistical analysis

Data was summarised using absolute numbers and percentages for categorical variables (gender) and median values with IQRs for continuous variables (seconds). Χ^2^ tests were used to assess differences between groups. Differences in speaking time between genders were analysed with unpaired t-tests. Data analysis was conducted using R software (V.4.2.3, R Foundation for Statistical Computing, Vienna, Austria) using the packages dplyr, tidyr, ggplot2 and gtsummary.

### Patient and public involvement

Patients and members of the public were not involved in the design, conduct, reporting or dissemination plans of this research.

## Results

We analysed 152 recorded sessions of the ASCO 2024 congress. Session types included 84 (55%) education sessions, 25 (16%) oral abstract sessions, 24 (16%) rapid oral abstract sessions, 16 (11%) clinical science symposia and 1 (<1%) award lecture, opening session and plenary session each. Session tracks are available in online supplemental table 1. There were 4 (3%) sessions without any recorded questions. In 90 (61%) of sessions with questions, there were only audio recordings of questioners, while 58 (39%) also contained video footage.

### Gender of chairs and speakers

**Figure 1:**
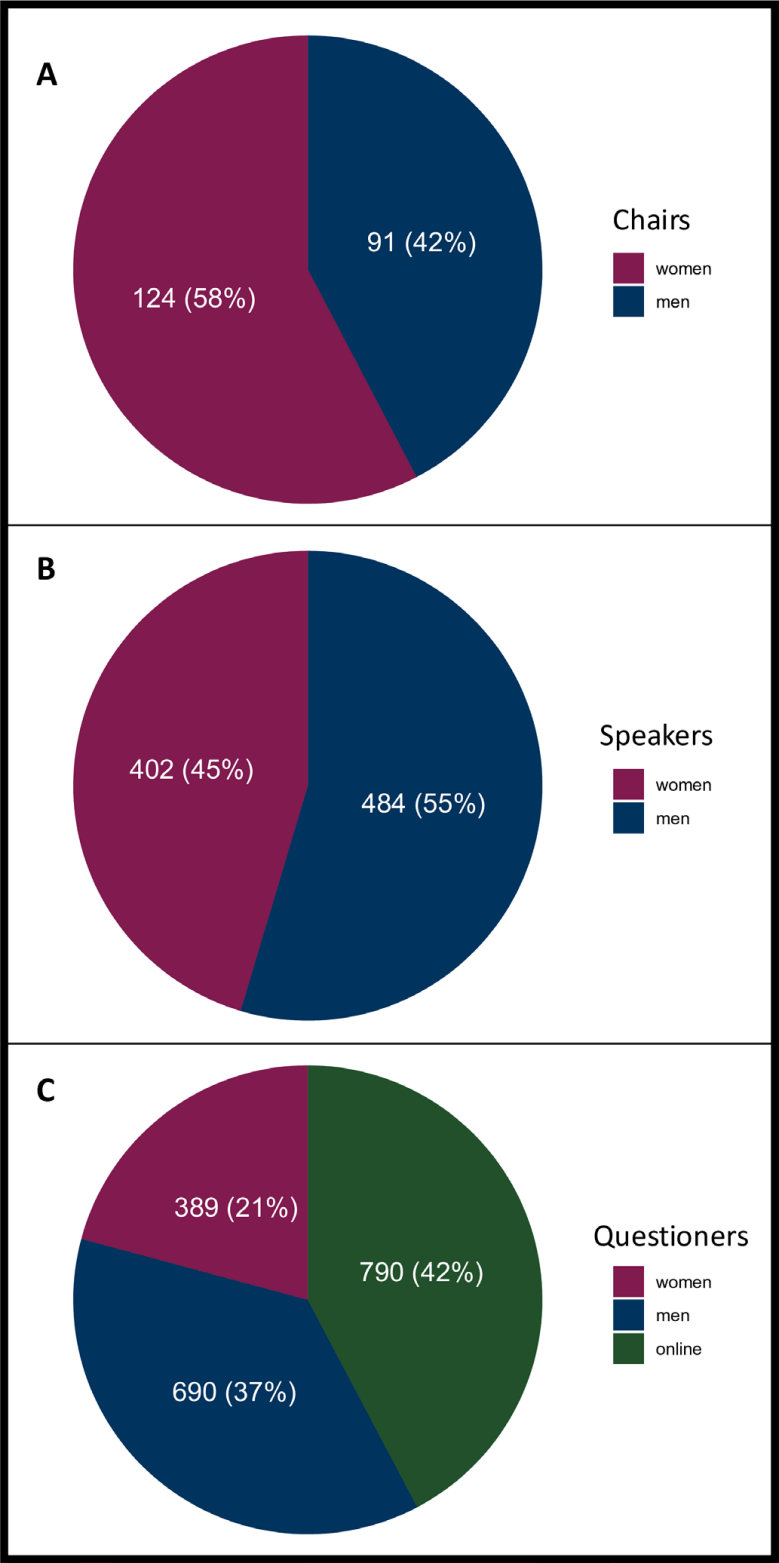
Gender distribution of chairs (**A**), speakers (**B**) and questioners (**C**).

Gender distribution of chairs and speakers is visualised in figure 1. There were 124 women who chaired in 101 (66%) sessions (78 with 1 woman, 23 with 2 women) and 91 men who chaired in 87 (57%) sessions (83 with 1 man, 4 with 2 men). Also, 37 (30%) women and 42 (46%) men were chairs as well as speakers of the respective sessions.

145 (95%) of sessions had at least one woman speaker, while 139 (91%) had at least one man. In total, there were 402 (45%) women and 484 (55%) men who contributed as a speaker. The number of chairs and speakers per session based on gender is available in table 1.

**Table 1:** Count of chairs and speakers per session based on gender

Variable	N (%)
Chairs - women	
0	51 (34)
1	78 (51)
2	23 (15)
Chairs - men	
0	65 (43)
1	83 (55)
2	4 (2.6)
Speakers - women	
0	7 (4.6)
1	41 (27)
2	39 (26)
3	30 (20)
4	11 (7.2)
5	9 (5.9)
6	5 (3.3)
7	8 (5.3)
9	2 (1.3)
Speakers - men	
0	13 (8.6)
1	29 (19)
2	41 (27)
3	12 (7.9)
4	14 (9.2)
5	14 (9.2)
6	9 (5.9)
7	9 (5.9)
8	5 (3.3)
9	6 (3.9)

### Genders of questioners

In total, 1869 questions and comments were recorded. Questions and comments from the audience were asked by women in 389 (21%) of cases and by men in 690 (37%) of cases. In addition, 790 (42%) questions were asked by chairs or other speakers, which represents questions posed online as well as discussions between the podium members. The distribution is visualised in figure 1.

**Figure 2:**
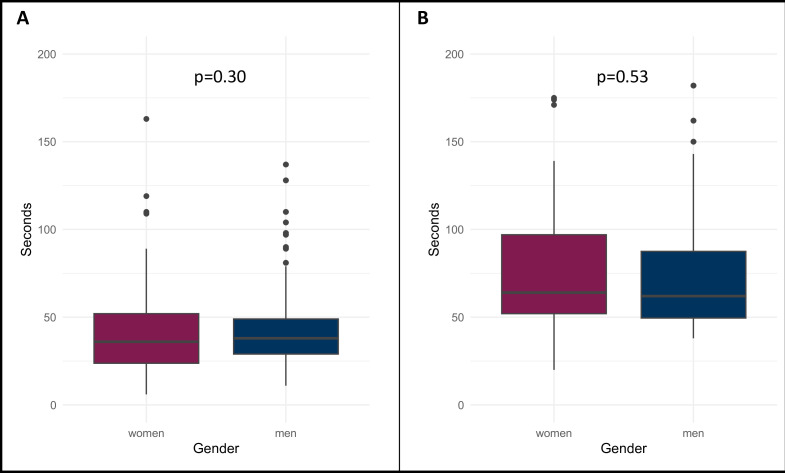
Boxplots showing median speaking time of women and men concerning (**A**) questions from the audience and (**B**) responses by speakers.

Women asked questions in 116 (76%) sessions, while men asked questions in 132 (86%) sessions. The median duration of questions was 36 (IQR 24–52) seconds for women, 38 (29–49) seconds for men and 18 (13–24) seconds for questions posed by chairs or other speakers. There was no statistically significant difference concerning speaking time between women and men posing questions (p=0.30), which is visualised in figure 2.

Further analyses investigating the distribution of questioners depending on the gender of chairs and speakers showed that 82 of 116 sessions (71%) with women questioners had women chairs, compared with 19 of 36 sessions (53%) with no women questioners (p=0.047). The presence of women speakers in a session was not associated with audience questions being asked by women (p=0.67).

For men, there was no statistically significant association between the presence of male chairs or speakers and the likelihood of men posing questions. In sessions with men questioners, 75 of 132 (57%) had men chairs, compared with 12 of 20 (60%) sessions without men questioners (p=0.79). The presence of men speakers was also not associated with audience questions being asked by men (121 of 132 sessions (92%) with men questioners vs 18 of 20 (90%) without; p=0.68).

### Gender of respondents

There were 1841 responses to questions and comments recorded. Women responded to 659 (36%), men to 898 (49%) and both genders to 284 (15%) questions. If both genders answered, in 147 cases women answered first and in 137 cases men answered first.

Figure 2 displays who responded to questions from women in the audience, men in the audience and questions submitted online or asked by the chair or speaker. Questions/comments posed by women were most frequently answered by women speakers (43%), while questions posed by men and questions submitted online or asked by the chair or speaker were most frequently answered by men (56% and 47%, respectively).

**Figure 3:**
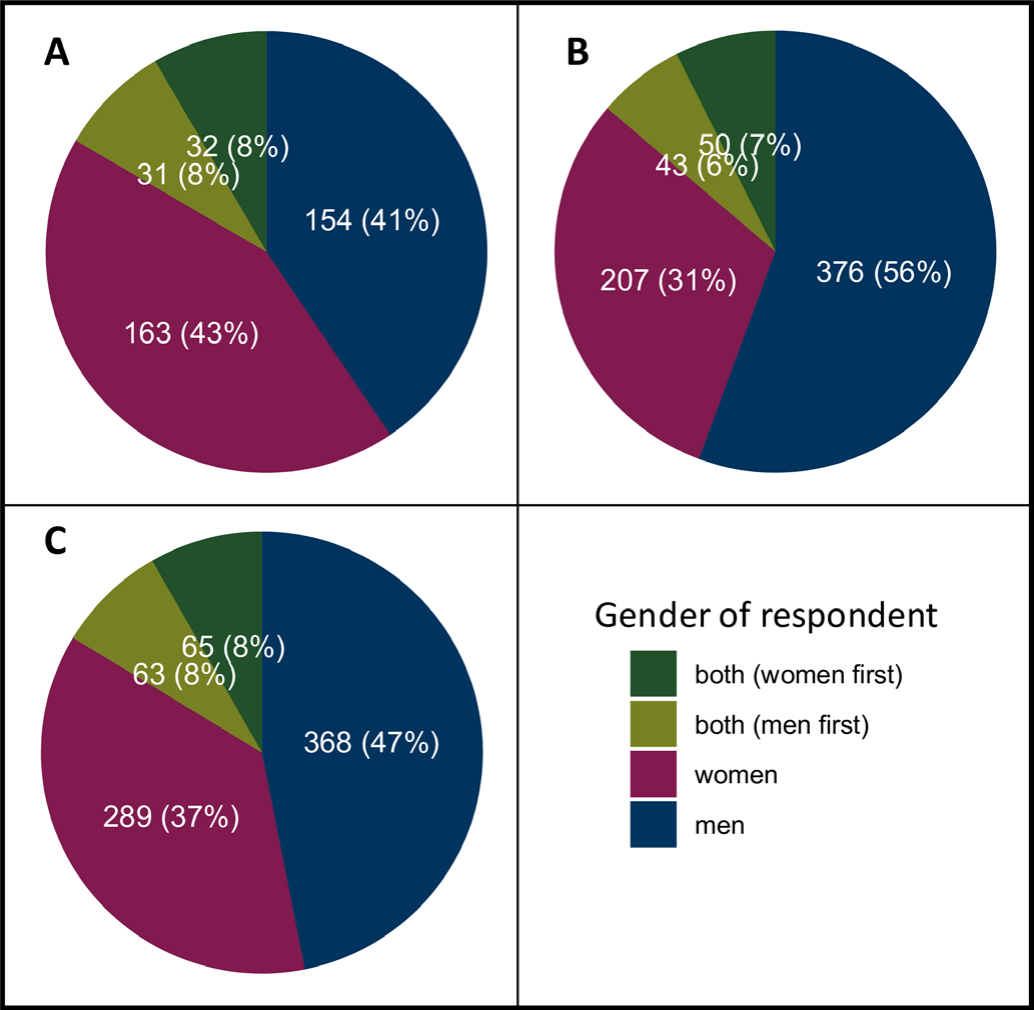
Gender response distribution to audience questions posed by women (**A**), men (**B**), and those submitted online or asked directly by the chair or speaker (**C**). Each colour represents the gender of the respective respondents. If a question was answered by speakers from both genders, it was stated which gender responded first.

Sessions where women asked questions saw a higher incidence of women answering (109 sessions, 94%) compared with sessions without women posing questions (30 sessions, 83%) (p=0.080). In addition, men answered questions more often in sessions where men posed questions (118 sessions, 89%) than in sessions where no men asked questions (14 sessions, 70%) (p=0.028).

The median time of responses from speakers was 64 (IQR 52–97) seconds for women and 62 (IQR 50–88) for men. There was no statistically significant difference (p=0.53), which is visualised in figure 2.

## Discussion

The ASCO 2024 congress showed considerable effort to achieve gender parity among chairs and speakers, which is in line with recent data of other large congresses.^17 19^ Considering prior ASCO congresses as well as prior medical conferences across other specialties, this emphasises the ongoing commitment of scientific societies to improve the representation of women.^11 20^

Despite these improvements at the leadership level at conferences, the broader professional environment in academic oncology remains shaped by longstanding gender-biased structures. Women oncologists frequently work in settings where they are under-represented in academic leadership roles, face implicit bias and encounter fewer mentorship opportunities and recognition.^5 9 21 22^ These systemic factors create barriers that may influence visibility and participation, including during conference discussions.

This structural context is reflected in our results, where a discrepancy persists in audience engagement, with questions posed by men overshadowing those of women (37% vs 21%). This imbalance in participation has also been reported for other conferences and could be caused by women being more likely to hold back questions because of anxiety, which, in turn, may have different reasons.^13 16^ For example, a previous study suggested that women ask less questions at conferences as they often have experienced discrimination or harassment at conferences before.^23^ Also, women often experience higher rates of self-doubt regarding their capabilities, which can lead to a reluctance to ask questions for fear that their inquiries may be perceived as naive or uninformed. The so-called impostor syndrome is a psychological pattern characterised by an inability to internalise personal achievements and a pervasive fear of being exposed as a ‘fraud’, which can significantly hinder professional confidence and career advancement. It is known that women as physicians are more likely to experience impostor syndrome than their male colleagues regardless of specialty or leadership role.^24 25^ In a recent survey, female gender was the only factor associated with higher impostor syndrome scores in medical oncologists.^26^ The prevalence of imposter syndrome among women underscores the psychological barriers that further complicate the professional landscape for women in oncology.

Our findings suggest that disparity in audience participation might be overcome by having women as chairs in congress sessions. Specifically, the probability of participation of women in posing questions was almost 20% higher in sessions with women as chairs. This suggests that women in leadership roles might foster a more supportive or relatable environment, encouraging gender diversity during academic discussions. Reasons behind this may stem from increased confidence among women in the audience to pose questions when the chair is also a woman or a tendency of women as chairs to call upon women at the microphones. This effect was also observed at conferences in other medical fields. For example, during the 2018 UK Society of Endocrinology’s meeting, it was requested that more women attended as chairs and chairs were invited to offer the opening question to a woman from the audience if there was the option to do so. This simple intervention resulted in improved conference participation of women.^14^ Similar effects are observed in training programs, where women physicians in leadership positions were associated with a higher percentage of women faculty, which insinuates this effect is translatable to other areas of academia.^21^

In our analysis, the presence of women in speaking roles in sessions was not associated with an increased frequency of questions by women from the audience, which might be explained by the already high number of sessions with speakers who are women (95% of all sessions). However, our analysis did indicate that women who posed questions might be more likely to receive responses from other women, and similarly, men’s questions were predominantly answered by other men. This pattern suggests that participants may feel more comfortable when interacting with peers of the same gender. Thus, gender homogeneity may play a major role in science communication and lead to unconscious biases, which could affect academic discourse. Further analyses are needed to understand the underlying factors and potential advantages and disadvantages of this phenomenon.^27^

Importantly, we observed no disparity in the duration of speaking, neither in posing questions nor responding to them, suggesting that when women choose to participate, they do so as assertively as men. This observation stands in contrast to other data from large congresses and might again reflect the efforts of the chairs to inclusively engage all participants equitably.^12 14^

Strengths and limitations of our analysis have to be addressed.

The ASCO 2024 annual meeting was chosen for this analysis because it is one of the largest oncology meetings worldwide and provides accessible video recordings of sessions including audience questions. This accessibility allowed for a comprehensive and unbiased analysis of real-time audience engagement, which would not have been feasible at most other conferences due to lack of available recordings. As stated in the methods section, the attribution of gender was based on audio and visual materials rather than self-identification by the individuals involved. This approach, while being limited, is consistent with prior research in the absence of self-reported gender data.^12 13 16 17^ In order to improve the accuracy of gender as perceived by observers, the recordings were investigated by two independent reviewers, with additional oversight by a third. Thus, the reliability of gender attributions by observers was enhanced and our analysis likely reflects how gender is perceived by other audience members. However, the bias of differences between self-perception and external assessment remains. Thus, the gender allocation in this analysis may not accurately correspond to the individual’s self-identified gender and, therefore, introduces ethical concerns related to misclassification. Furthermore, gender was assigned only as either woman or man, and no other gender identities were allocated. This presents another limitation and ethical concern, as other gender identities may have been present but could not be identified due to the absence of self-reported data. These limitations highlight the need for future studies to incorporate self-reported demographic data where possible.

Additionally, the lack of data regarding the gender distribution of congress attendees prevents a direct comparison between the proportion of attendees and the proportion of active participants, limiting a full understanding of gender dynamics within the congress. Public availability of data on the gender, ethnicity and educational or professional background of congress attendees would substantially strengthen the contextual interpretation of participation metrics. Our findings underscore the importance of increased transparency and systematic reporting of demographic information in future congresses.

Another limitation that has to be addressed is that, in some sessions, questions were directed to specific speakers, while in others they were posed to the panel as a whole. However, it was often not possible to determine the intended recipient of each question based on the available video and audio recordings. As a result, the outcome ‘gender of respondents’ refers only to the individual who answered the question, not necessarily the person to whom it was directed. This may limit the interpretation of whether certain genders were more likely to be addressed by the audience. Furthermore, this study is based on the analysis of a single conference, which may limit the generalisability of the findings. However, this study represents an initial step towards increasing attention to attendee participation and gender dynamics at large oncological congresses. Future analyses that include multiple conferences may also allow for detailed subgroup analyses (ie, disparities between specific session types and tracks), which were not feasible in this setting due to limited data.

In conclusion, while the balanced representation of women in chair and speaker roles at ASCO 2024 reflects a positive development in gender equality, our findings highlight the ongoing need for strategies that encourage active participation of women in scientific dialogues. The presence of women in leadership roles is a key element to promote engagement of women, suggesting that future congresses should continue to focus on enhancing the visibility of women.

## Supplementary material

10.1136/bmjopen-2025-104821 online supplemental file 1

## Data Availability

Data are available in a public, open access repository. Data are available upon reasonable request.
