# Supplementary material for: Are we closing the gender gap in academic oncology? An observational study of gender disparities in participant engagement at the ASCO 2024 annual meeting
Source: BMJ Open. 2025 Sep 9;15(9):e104821. doi: 10.1136/bmjopen-2025-104821 (PMC12421591; doi:10.1136/bmjopen-2025-104821)
Supplement: online supplemental file 1 [file bmjopen-15-9-s001.pdf]

Variable	N (%)
**Session_Type**	
Award Lecture	1 (0.7%)
Clinical Science Symposium	16 (11%)
Education Session	84 (55%)
Opening Session	1 (0.7%)
Oral Abstract Session	25 (16%)
Plenary Session	1 (0.7%)
Rapid Oral Abstract Session	24 (16%)
**Session_Track**	
Breast Cancer	11 (7.2%)
Care Delivery and Quality Care	11 (7.2%)
Central Nervous System Tumors	6 (3.9%)
Developmental Therapeutics—Immunotherapy	5 (3.3%)
Developmental Therapeutics—Molecularly Targeted Agents and Tumor Biology	6 (3.9%)
Gastrointestinal Cancer—Colorectal and Anal	5 (3.3%)
Gastrointestinal Cancer—Gastroesophageal, Pancreatic, and Hepatobiliary	7 (4.6%)
Genitourinary Cancer—Kidney and Bladder	5 (3.3%)
Genitourinary Cancer—Prostate, Testicular, and Penile	6 (3.9%)
Gynecologic Cancer	6 (3.9%)
Head and Neck Cancer	5 (3.3%)
Hematologic Malignancies	16 (11%)
Lung Cancer	11 (7.2%)
Medical Education and Professional Development	10 (6.6%)
Melanoma/Skin Cancers	5 (3.3%)
Pediatric Oncology	6 (3.9%)
Prevention, Risk Reduction, and Genetics	6 (3.9%)
Sarcoma	6 (3.9%)
Special Sessions	13 (8.6%)
Symptom Science and Palliative Care	6 (3.9%)

Supplementary Table 1: Sessions type and session track of investigated sessions from the ASCO 2024 congress
