# Supplementary material for: Are we closing the gender gap in academic oncology? An observational study of gender disparities in participant engagement at the ASCO 2024 annual meeting
Source: BMJ Open. 2025 Sep 9;15(9):e104821. doi: 10.1136/bmjopen-2025-104821 (PMC12421591; doi:10.1136/bmjopen-2025-104821)
Supplement: Supplementary file 2 [file bmjopen-2025-104821.reviewer_comments.pdf]

PEER REVIEW HISTORY

BMJ Open publishes all reviews undertaken for accepted manuscripts. Reviewers are asked to complete a checklist review form and are provided with free text boxes to elaborate on their assessment. These free text comments are reproduced below.

ARTICLE DETAILS

Title (Provisional)

Are We Closing the Gender Gap in Academic Oncology? An Observational Study of Gender Disparities in Participant Engagement at the ASCO 2024 Annual Meeting

Authors

Puhr, Hannah Christina; Cammarota, A; Ettaieb, M; Flierman, Isabelle; Gisinger, T; Glas, A; Guven, Deniz C; Siebenhüner, Alexander; Steindl, A; Szydlik, V; Valpione, S; Yip, M; Laarhoven, Hanneke W. M. van

---

VERSION 1 - REVIEW

**Reviewer**	**1**
**Name**	**Wang , Betty**
**Affiliation**	**Cleveland Clinic, Urology**
**Date**	**01-Jul-2025**
**COI**	**None**

It would be valuable to provide data on the gender distribution of audience attendees, as this would help contextualize the proportion of questions asked by women. For example, if certain sessions had only 25% female attendees but 40% of the questions came from women, this might actually indicate higher relative engagement rather than underrepresentation. While the authors acknowledge this limitation, explicitly including these data in future analyses would strengthen the interpretation.

Additionally, not all sessions hold the same weight at large conferences. It would be insightful to analyze "high-impact" sessions separately—such as plenary sessions, state-of-the-art lectures, or invited keynote talks—which typically draw larger audiences and feature prominent speakers. Gender representation among chairs, speakers, and questioners in these sessions may have greater implications for visibility and influence.

Finally, although the methodology is innovative and well executed, analyzing a single time point (ASCO 2024) may limit the conclusions. Including data from other major meetings in the same year (e.g., ESMO) or extending the analysis across multiple years of ASCO would allow for temporal trend analysis and improve the robustness and generalizability of the findings.

**Reviewer**	**2**
**Name**	**Millet, Nessa**
**Affiliation**	**Amsterdam UMC Location VUmc, Medical Psychology**

Date **16-Jul-2025**

COI **None**

Are We Closing the Gender Gap in Academic Oncology? Gender Disparities in Conference Participant Engagement- review comments

Summary
Thank you for the opportunity to review this manuscript. The study provides some interesting data on the gender differences in participation at the American Society for Clinical Oncology conference, however these are mostly limited to descriptive data in gender differences in chairing and speaking along with question posing. The authors lay out a compelling argument for conducting this work, given the evident gender disparities in oncology and the impact of gender on career prospects. However, the conclusions that can be made from this study are limited, and I feel that the current discussion points are over reaching. There are also areas where increased clarity on the methods are necessary to enhance readership. Please see my detailed feedback below:

General

The outcomes of this study are the gender of chairs, speakers, people who posed questions, people who responded to questions and the time spent of speaking. Whilst this data is insightful, in particular the data on differences in numbers of questions posed by men and women, I believe that stronger and more in depth data would be beneficial to bolster any conclusions that can inform strategies for engaging women in scientific discussions. In the study discussion, the authors make hypothetical claims to explain their results which can be seen elsewhere in the literature, but which are generally weakly supported (e.g., women self-doubting their abilities compared to men, the role of gender homogeneity in science communication, and unconscious bias) and feel quite reductionist, particularly given that the authors do not measure sociodemographic characteristics (e.g., ethnicity, education level) which could also feasibly influence such processes. These mechanisms would be better explained using a study design that uses a mixed methods approach where the experiences of participants attending oncology conferences can be explored and used to explain quantitative data. Did the authors consider this approach, and would it be possible to collect retrospective qualitative data to bolster these findings?

Abstract
The abstract is clear and informative.

Introduction
There is a mix of using 'female' and 'women' in the introduction. In line with guidance on reporting sex and gender in research (Sex and Gender Equity in Research: rationale for the SAGER guidelines and recommended use | Research Integrity and Peer Review | Full Text) can the authors please define the concept of gender in their study and consistently refer to this throughout?

Page 7, Line 19- Can you outline some examples of initiatives, as this links back to one of the key findings about strategies to promote involvement of women?

Method

The main outcome of 'gender' relies on the researcher's own observation of participant characteristics such as appearance, names and voice characteristics. In this approach, participant's gender is being reduced to gender categories of man and woman and therefore ignoring other possible gender identities. The authors do not support this approach with previous research, and fail to fully reflect on how their own position may influence their appraisal, or the ethical implications of this for participants.

Given that this study concerns gender parity, I believe this section requires further elaboration on potential ethical considerations and explanation of the method of assignment, for example:

- Can the authors please provide literature or methods to support this approach to assigning gender in this way and specify in more detail the characteristics on which their assignment is based? Relatedly I would urge the authors to include a statement acknowledging that the gender they assign to someone may not reflect that person's felt gender identity (Please see for example: Women's visibility in academic seminars: Women ask fewer questions than men - PMC)

- Can the authors also add information on how this type of approach bolsters their study design. For example, are the authors also interested in how a participant is perceived by other attendees (e.g., as masculine or feminine) and therefore, is the assigning of 'perceived gender' instrumental in this?

- Can the authors please provide a reflexive account of how they believe their own positions and perspectives influence their appraisal of participant's gender, and comment on the ethical complications of this type of data collection?

The outcome of 'gender responder' is not clear to me. In many conference settings, questions are asked to a particular speaker about their work, rather than being asked to a large group of people. Thus, does this outcome actually measure the tendency for someone to ask a question to a certain gender rather than the gender of the respondent, as this would be pre-determined if the question is aimed at a particular person? Please clarify.

Discussion

It seems that the discussion starts with framing the gender disparities in leadership and congress participation using a predominantly woman deficit perspective (e.g., women being anxious, having more self-doubt, having imposter syndrome, experiencing psychological barriers). The authors do allude to the multidimensional nature of this problem (page 14, line 19) however, I believe the authors could do more to set the scene for these explanations. As it stands, it seems like there is an important piece on the historical and institutional gendered context in which these complex dynamics happen that is missing. For example, can the authors explain to other readers what it is like to be a woman oncologist working in such environments, and better explain the nature of such settings? This would then lead into the existing explanatory points in a more balanced way.

Page 15, lines 13- 20. The discussion point on whether women and men are more likely to respond to questions posed by the same perceived gender relate to my question regarding this outcome in the methods. This discussion points seems redundant if in fact a question was purposefully posed to someone of the same perceived gender. Can the authors please clarify?

VERSION 1 - AUTHOR RESPONSE

Reviewer #1:

Dr. Betty Wang , Cleveland Clinic

Response:
Thank you for reading our manuscript. We sincerely appreciate your thoughtful and constructive comments. Below, we address each point in a point-by-point format.

Comments to the Author:
It would be valuable to provide data on the gender distribution of audience attendees, as this would help contextualize the proportion of questions asked by women. For example, if certain sessions had only 25% female attendees but 40% of the questions came from women, this might actually indicate higher relative engagement rather than underrepresentation. While the authors acknowledge this limitation, explicitly including these data in future analyses would strengthen the interpretation.

Response:
Thank you for addressing this crucial point. Unfortunately, attendance data for the congress is not publicly available, and multiple attempts to obtain this information directly from ASCO were unsuccessful. A query to the CENTRA team of ASCO (https://www.asco.org/research-data/asco-data-library), which is responsible for data management, confirmed that the information is not available publicly and, thus, cannot be shared.
We had already mentioned this limitation in the manuscript. However, in response to the reviewer's comment, we have revised the relevant section to emphasize this point more clearly.
We added the section:
"Public availability of data on the gender, ethnicity, and educational or professional background of congress attendees would substantially strengthen the contextual interpretation of participation metrics. Our findings underscore the importance of increased transparency and systematic reporting of demographic information in future congresses."

Comment:
Additionally, not all sessions hold the same weight at large conferences. It would be insightful to analyze "high-impact" sessions separately—such as plenary sessions, state-of-the-art lectures, or invited keynote talks—which typically draw larger audiences and feature prominent speakers. Gender representation among chairs, speakers, and questioners in these sessions may have greater implications for visibility and influence.

Response:
Thank you for this insightful comment. We agree that analyzing high-impact sessions separately—such as plenary or keynote lectures—could provide valuable insight into gender representation in the most visible and influential settings. However, there is currently no universally accepted standard for classifying the relative importance of sessions. For the purposes of this study, session types were categorized according to ASCO's official classification, as outlined in Supplementary Table 1. Only a small number of sessions (e.g., plenary session, opening session, award lecture) fall into the "high-impact" category, which would have resulted in very limited data for subgroup analysis.

Nonetheless, this is an excellent suggestion and will be considered in future analyses involving additional congresses. We have added a corresponding note to the Limitations section.
"Future analyses that include multiple conferences may also allow for detailed subgroup analyses (i.e. disparities between specific session types and tracks), which were not feasible in this setting due to limited data."

Comment:
Finally, although the methodology is innovative and well executed, analyzing a single time point (ASCO 2024) may limit the conclusions. Including data from other major meetings in the same year (e.g., ESMO) or extending the analysis across multiple years of ASCO would allow for temporal trend analysis and improve the robustness and generalizability of the findings.

Response:
We appreciate this valuable comment. Expanding the analysis to include multiple congresses would indeed provide a more comprehensive perspective. However, comparable data from ESMO congresses were not available, as those meetings do not systematically record or archive audience comments. Plans to extend this work to other ASCO congresses are under consideration, and the current study is intended as an initial step toward a broader evaluation of participation patterns across oncology conferences. We added a sentence to emphasize this: "Furthermore, this study is based on the analysis of a single conference, which may limit the generalizability of the findings. However, this study represents an initial step toward increasing attention to attendee participation and gender dynamics at large oncological congresses."

Reviewer #2:

Dr. Nessa Millet, Amsterdam UMC Location VUmc, University of Leicester

Comment:
Summary
Thank you for the opportunity to review this manuscript. The study provides some interesting data onnthe gender differences in participation at the American Society for Clinical Oncology conference,nhowever these are mostly limited to descriptive data in gender differences in chairing and speaking along with question posing. The authors lay out a compelling argument for conducting this work, given the evident gender disparities in oncology and the impact of gender on career prospects. However, the conclusions that can be made from this study are limited, and I feel that the current discussion points
are over reaching. There are also areas where increased clarity on the methods are necessary to enhance readership. Please see my detailed feedback below:

Response:
We sincerely appreciate the time and effort you dedicated to reviewing our manuscript. Your comments were insightful and constructive, and we believe the revisions made in response have significantly improved the quality and clarity of the work. Please find our detailed, point-by-point responses below.

Comment:
General
The outcomes of this study are the gender of chairs, speakers, people who posed questions, people who responded to questions and the time spent of speaking. Whilst this data is insightful, in particular the data on differences in numbers of questions posed by men and women, I believe that stronger and more in depth data would be beneficial to bolster any conclusions that can inform strategies for engaging women in scientific discussions. In the study discussion, the authors make hypothetical claims to explain their results which can be seen elsewhere in the literature, but which are generally weakly supported (e.g., women self-doubting their abilities compared to men, the role of gender homogeneity in science communication, and unconscious bias) and feel quite reductionist, particularly
given that the authors do not measure sociodemographic characteristics (e.g., ethnicity, education level) which could also feasibly influence such processes. These mechanisms would be better explained using a study design that uses a mixed methods approach where the experiences of participants attending oncology conferences can be explored and used to explain quantitative data.

Did the authors consider this approach, and would it be possible to collect retrospective qualitative data to bolster these findings?

Response:
Thank you for this valuable insight. We fully agree that more detailed demographic data would enhance the interpretation and depth of the findings. Initially, an attempt was made to include variables such as ethnicity and educational background. However, this proved infeasible, as the majority of audience members do not provide identifying information such as name, affiliation, or professional background. Efforts were made to obtain aggregate demographic data from ASCO, but these were unsuccessful despite multiple attempts. A query to the CENTRA team of ASCO (https://www.asco.org/research- data/asco-data-library), which is responsible for data management, confirmed that the information is not available publicly and, thus, cannot be shared. To address this limitation more clearly, we have added a corresponding statement to the limitations section of the manuscript.
We added this to our Discussion section:
"Public availability of data on the gender, ethnicity, and educational or professional background of congress attendees would substantially strengthen the contextual interpretation of participation metrics. Our findings underscore the importance of increased transparency and systematic reporting of demographic information in future congresses."

Comment:
Abstract
The abstract is clear and informative.

Response:
We appreciate this positive feedback.

Comment:
Introduction
There is a mix of using 'female' and 'women' in the introduction. In line with guidance on reporting sex and gender in research (Sex and Gender Equity in Research: rationale for the SAGER guidelines and recommended use | Research Integrity and Peer Review | Full Text) can the authors please define the concept of gender in their study and consistently refer to this throughout?

Response:
Thank you for this remark. In the respective sections of the Background and Discussion, we initially used the term female to remain consistent with the wording of the referenced literature. However, in our own analysis, we consistently used the term woman/women.
We agree that consistent terminology throughout the manuscript improves clarity and readability. Therefore, we have revised the manuscript to use woman/women uniformly, which we believe enhances coherence and avoids potential confusion.
We also added a sentence and reference that this is in line with the AMA guidelines: "Gender terminology in this study aligns with AMA guidelines[12]."

Comment:
Page 7, Line 19- Can you outline some examples of initiatives, as this links back to one of the key findings about strategies to promote involvement of women?

Response:
We appreciate this remark and put some examples with links to their webpages in our

manuscript:
"e.g. Leadership programme for Women in Oncology (LPWO), Women Who Conquer Cancer (WWCC), ESMO Women for Oncology (W4O)"

Comment:
Method
The main outcome of 'gender' relies on the researcher's own observation of participant characteristics such as appearance, names and voice characteristics. In this approach, participant's gender is being reduced to gender categories of man and woman and therefore ignoring other possible gender identities. The authors do not support this approach with previous research, and fail to fully reflect on how their own position may influence their appraisal, or the ethical implications of this for participants. Given that this study concerns gender parity, I believe this section requires further elaboration on potential ethical considerations and explanation of the method of assignment, for example:

- Can the authors please provide literature or methods to support this approach to assigning gender in this way and specify in more detail the characteristics on which their assignment is based? Relatedly I would urge the authors to include a statement acknowledging that the gender they assign to someone may not reflect that person's felt gender identity (Please see for example: Women's visibility in academic seminars: Women ask fewer questions than men - PMC)
- Can the authors also add information on how this type of approach bolsters their study design. For example, are the authors also interested in how a participant is perceived by other attendees (e.g., as masculine or feminine) and therefore, is the assigning of 'perceived gender' instrumental in this?
- Can the authors please provide a reflexive account of how they believe their own positions and perspectives influence their appraisal of participant's gender, and comment on the ethical complications of this type of data collection?

Response:
Thank you for this important and thoughtful comment. We acknowledge the limitations inherent in assigning gender based on perceived characteristics and agree that accurate methodological transparency is essential.
Our method to determine gender based on visual and auditory characteristics from the video recordings, such as appearance, names, and voice characteristics are common practice in observational studies (Carter, Croft et al. 2018, Davids, Lyu et al. 2019, Jarvis, Ebersole et al. 2022, Rezaee, Verde et al. 2022).
We added more precisely how we determined appearance and voice characteristics: "Gender was defined as the categorization (woman, man) perceived by the reviewers based on visual and auditory characteristics from the video recordings, such as names (i.e. etymology and/or publicly available professional profiles), appearance (i.e. attire, hairstyle, use of makeup), and voice characteristics (i.e. pitch and tone)."
While imperfect, this practice is a common and pragmatic solution when demographic data are not available. The assignment of perceived gender was not intended to analyze gender presentation (e.g. masculinity or femininity) or social perception, but was used solely as a practical approach to enable gender-based analysis in the absence of self- reported data. To limit potential bias and ensure consistency, gender was initially assessed independently by two reviewers. A third reviewer then re-evaluated all assignments, specifically focusing on cases of potential discrepancy. Very few differences were identified, and any inconsistencies were easily resolved through review and discussion.
However, ethical concerns corresponding to self-identification remain as a limitation.
We already discussed this approach as a limitation in our Discussion section and based on your

suggestions extended this section to emphasize it further. We also added several references where similar approaches were used:
"As stated in the methods section, the attribution of gender was based on audio and visual materials rather than self-identification by the individuals involved. This approach, while being limited, is consistent with prior research in the absence of self-reported gender data[12,13,16,21]. In order to improve the accuracy of gender as perceived by observers, the recordings were investigated by two independent reviewers, with additional oversight by a third. Thus, reliability of gender attributions by observers was enhanced and our analysis likely reflects how gender is perceived by other audience members. However, the bias of differences between self-perception and external assessment remains. Thus, the gender allocation in this analysis may not accurately correspond to the individual's self-identified gender and, therefore, introduces ethical concerns related to misclassification.
Furthermore, gender was assigned only as either woman or man, and no other gender identities were allocated. This presents another limitation and ethical concern, as other gender identities may have been present but could not be identified due to the absence of self-reported data. These limitations highlight the need for future studies to incorporate self-reported demographic data where possible."

We also like to thank you for the example you provided. This publication underlines our findings and we added it as a reference to our Discussion section.

Comment:
The outcome of 'gender responder' is not clear to me. In many conference settings, questions are asked to a particular speaker about their work, rather than being asked to a large group of people. Thus, does this outcome actually measure the tendency for someone to ask a question to a certain gender rather than the gender of the respondent, as this would be pre-determined if the question is aimed at a particular person? Please clarify.

Response:
Thank you for this thoughtful observation. We acknowledge that in some sessions, questions were directed to the entire panel, while in others they were clearly addressed to a specific speaker. Unfortunately, it was not feasible to reliably capture this distinction based on the available video and audio recordings. In many cases, the intended target of the question was ambiguous or only inferable through detailed content analysis, which was not possible in this retrospective online format. As such, our outcome of "gender of the responder" reflects who responded to the question rather than to whom the question was necessarily directed. This limitation has now been added to the manuscript.
"Another limitation that has to be addressed is that, in some sessions, questions were directed to specific speakers, while in others they were posed to the panel as a whole. However, it was often not possible to determine the intended recipient of each question based on the available video and audio recordings. As a result, the outcome "gender of respondents" refers only to the individual who answered the question, not necessarily the person to whom it was directed. This may limit the interpretation of whether certain genders were more likely to be addressed by the audience."

Comment:
Discussion
It seems that the discussion starts with framing the gender disparities in leadership and congress participation using a predominantly woman deficit perspective (e.g., women being anxious, having more self-doubt, having imposter syndrome, experiencing psychological barriers). The authors do allude to the multidimensional nature of this problem (page 14, line

19) however, I believe the authors could do more to set the scene for these explanations. As it stands, it seems like there is an important piece on the historical and institutional gendered context in which these complex dynamics happen that is missing. For example, can the authors explain to other readers what it is like to be a woman oncologist working in such environments, and better explain the nature of such settings? This would then lead into the existing explanatory points in a more balanced way.

Response:
We appreciate this comment and added a section at the beginning of the discussion to address the broader systemic context.
"Despite these improvements at the leadership level at conferences, the broader professional environment in academic oncology remains shaped by longstanding gender biased structures. Women oncologists frequently work in settings where they are underrepresented in academic leadership roles, face implicit bias, and encounter fewer mentorship opportunities and recognition[5,9,19,20]. These systemic factors create barriers that may influence visibility and participation, including during conference discussions. This structural context is reflected in our results, where a discrepancy persists in audience engagement, with questions posed by men overshadowing those of women (37% vs. 21%)."

Comment:
Page 15, lines 13-20. The discussion point on whether women and men are more likely to respond to questions posed by the same perceived gender relate to my question regarding this outcome in the methods. This discussion points seems redundant if in fact a question was purposefully posed to someone of the same perceived gender. Can the authors please clarify?

Response:
As stated above we added a paragraph in the Limitations section to elaborate on this issue.

VERSION 2 - REVIEW

**Reviewer**	**1**
**Name**	**Wang , Betty**
**Affiliation**	**Cleveland Clinic, Urology**
**Date**	**15-Aug-2025**
**COI**	

The authors have adequately addressed both my comments and those of reviewer #2, I believe the paper may be accepted as is, or with minor revisions per review #2 or the editorial team. Thank you

**Reviewer**	**2**
**Name**	**Millet, Nessa**
**Affiliation**	**Amsterdam UMC Location VUmc, Medical Psychology**

Date **13-Aug-2025**

COI

The authors have responded to most previous comments and adjusted the manuscript accordingly. In particular there is increased clarity in the methods and stronger substantiation in the discussion. I have some further points which would add increased clarity for the readership of the manuscript:

Abstract

Outlining the ‘participants’ section as 'Gender for chairs, speakers, and audience questioners were evaluated' seems more like the data collection. Can the authors please rewrite to reflect the participant group (i.e. people who participated in certain sessions at the ASCO conference)

Methods

You have included supporting methodological examples for the method used to assign gender in the strengths and limitations section (This approach, while being limited, is consistent with prior research in the absence of self-reported gender data12,13,17,22). However, I believe that this would be better placed in the methods section where you outline the gender outcomes so that readers are aware of this method from the onset

As previously suggested, please can the authors include a statement acknowledging hat the gender assigned to attendees may not align with attendee’s own gender identity.

Results

The following sentence is not possible to follow: For men, there was no statistically significant association with men chairs (12/20 (60%) sessions without and 75/132 (57%) sessions with questions posed by men; p=0.79) or men as speakers (18/20 (90%) and 121/132 (92%) sessions; p=0.68). Can the authors please re-word to outline between what variables the association was measured?

VERSION 2 - AUTHOR RESPONSE

Reviewer: 1

Comment:
Comments to the Author:
The authors have adequately addressed both my comments and those of reviewer #2, I believe the paper may be accepted as is, or with minor revisions per review #2 or the editorial team. Thank you

Response:
Thank you very much for reviewing our manuscript.

Reviewer: 2

Comment:

Comments to the Author:

The authors have responded to most previous comments and adjusted the manuscript accordingly. In particular there is increased clarity in the methods and stronger substantiation in the discussion. I have some further points which would add increased clarity for the readership of the manuscript:

Abstract

Outlining the 'participants' section as 'Gender for chairs, speakers, and audience questioners were evaluated' seems more like the data collection. Can the authors please rewrite to reflect the participant group (i.e. people who participated in certain sessions at the ASCO conference)

> ***Response:***
> *Thank you for this comment. We revised the section, but could not elaborate further due to word count restrictions:*
> *"Participants:*
> *Individuals serving as chairs, speakers, and audience members who asked questions"*

Comment:

Methods

You have included supporting methodological examples for the method used to assign gender in the strengths and limitations section (This approach, while being limited, is consistent with prior research in the absence of self-reported gender data12,13,17,22). However, I believe that this would be better placed in the methods section where you outline the gender outcomes so that readers are aware of this method from the onset

> ***Response:***
> *We have now incorporated this point in our Methods section:*
> *"This method, though limited, is consistent with prior research12,13,16,17."*
> *However, we have also retained this point in the Discussion section, as we believe it is important to explicitly acknowledge and reflect on the limitations of this approach in the context of interpreting our findings.*

Comment:

As previously suggested, please can the authors include a statement acknowledging hat the gender assigned to attendees may not align with attendee's own gender identity.

> ***Response:***
> *Thank you for this suggestion. We have already included this point in our Discussion and now also added it to the Methods section:*

“It has to be acknowledged that the assigned gender may not necessarily correspond to attendees’ self-identified gender.”

Comment:

Results

The following sentence is not possible to follow: For men, there was no statistically significant association with men chairs (12/20 (60%) sessions without and 75/132 (57%) sessions with questions posed by men; p=0.79) or men as speakers (18/20 (90%) and 121/132 (92%) sessions; p=0.68). Can the authors please re-word to outline between what variables the association was measured?

Response:

We appreciate this comment.

We adapted the section:

“For men, there was no statistically significant association between the presence of male chairs or speakers and the likelihood of men posing questions. In sessions with men questioners, 75 of 132 (57%) had men chairs, compared to 12 of 20 (60%) sessions without men questioners (p = 0.79). The presence of men speakers was also not associated with audience questions being asked by men (121 of 132 sessions (92%) with men questioners vs. 18 of 20 (90%) without; p = 0.68).”
